# Bayesian Estimation of Mixture Models with Prespecified Elements to Compare Drug Resistance in Treatment-Naïve and Experienced Tuberculosis Cases

**DOI:** 10.1371/journal.pcbi.1002973

**Published:** 2013-03-21

**Authors:** Alane Izu, Ted Cohen, Victor DeGruttola

**Affiliations:** 1 Department of Science and Technology/National Research Foundation: Vaccine Preventable Diseases and Respiratory & Meningeal Pathogens Research Unit, University of Witwatersrand, Faculty of Health Science, Johannesburg, Gauteng, South Africa; 2 Department of Epidemiology, Harvard School of Public Health, Boston, Massachusetts, United States of America; 3 Division of Global Health Equity, Brigham and Women's Hospital, Boston, Massachusetts, United States of America; 4 Department of Biostatistics, Harvard School of Public Health, Boston, Massachusetts, United States of America; University of New South Wales, Australia

## Abstract

We propose a Bayesian approach for estimating branching tree mixture models to compare drug-resistance pathways (i.e. patterns of sequential acquisition of resistance to individual antibiotics) that are observed among *Mycobacterium tuberculosis* isolates collected from treatment-naïve and treatment-experienced patients. Resistant pathogens collected from treatment-naïve patients are strains for which fitness costs of resistance were not sufficient to prevent transmission, whereas those collected from treatment-experienced patients reflect both transmitted and acquired resistance, the latter of which may or may not be associated with lower transmissibility. The comparison of the resistance pathways constructed from these two groups of drug-resistant strains provides insight into which pathways preferentially lead to the development of multiple drug resistant strains that are transmissible. We apply the proposed statistical methods to data from worldwide surveillance of drug-resistant tuberculosis collected by the World Health Organization over 13 years.

## Author Summary

Drug-resistant tuberculosis (TB) initially arises as a result of the sporadic appearance and subsequent selection of drug-resistant *M. tuberculosis* mutants. Such strains may or may not be associated with fitness costs affecting their ability to transmit and cause disease. Resistant pathogens collected from treatment-naïve patients are strains for which fitness costs of resistance were not sufficient to prevent transmission. Those collected from treatment-experienced patients reflect strains that may or may not be associated with lower transmissibility. Determining which strains are sufficiently fit to be transmitted and cause disease can aid in developing effective strategies to combat the spread of resistance. Branching trees are graphical models used to infer the sequence of several binary events (i.e. a pathway) that have occurred in an unknown order. We propose a novel method using branching trees with prespecified components to compare evolutionary pathways among different populations. We apply our model to understand if there are unique drug-resistant pathways found only amongst treatment experienced patients that might reflect acquired resistant disease associated with fitness costs that limits its ability to transmit. Our methods can be generalized to any biological process for which the assumption of an ascending markov process applies.

## Introduction

Tuberculosis (TB) is an infectious disease caused by *Mycobacterium tuberculosis* and is transmitted between hosts through the respiratory route. The appearance of TB resistant to multiple antibiotics threatens global control strategies that depend on the efficacy of standard combinations of these drugs. Drug-resistant TB in communities initially arises as a result of the sporadic appearance and subsequent selection of drug-resistant *M. tuberculosis* mutants in individuals receiving inadequate treatment. Individuals acquiring drug-resistance as a result of poor TB treatment may then transmit resistant organisms to their respiratory contacts.

**Figure 1:**
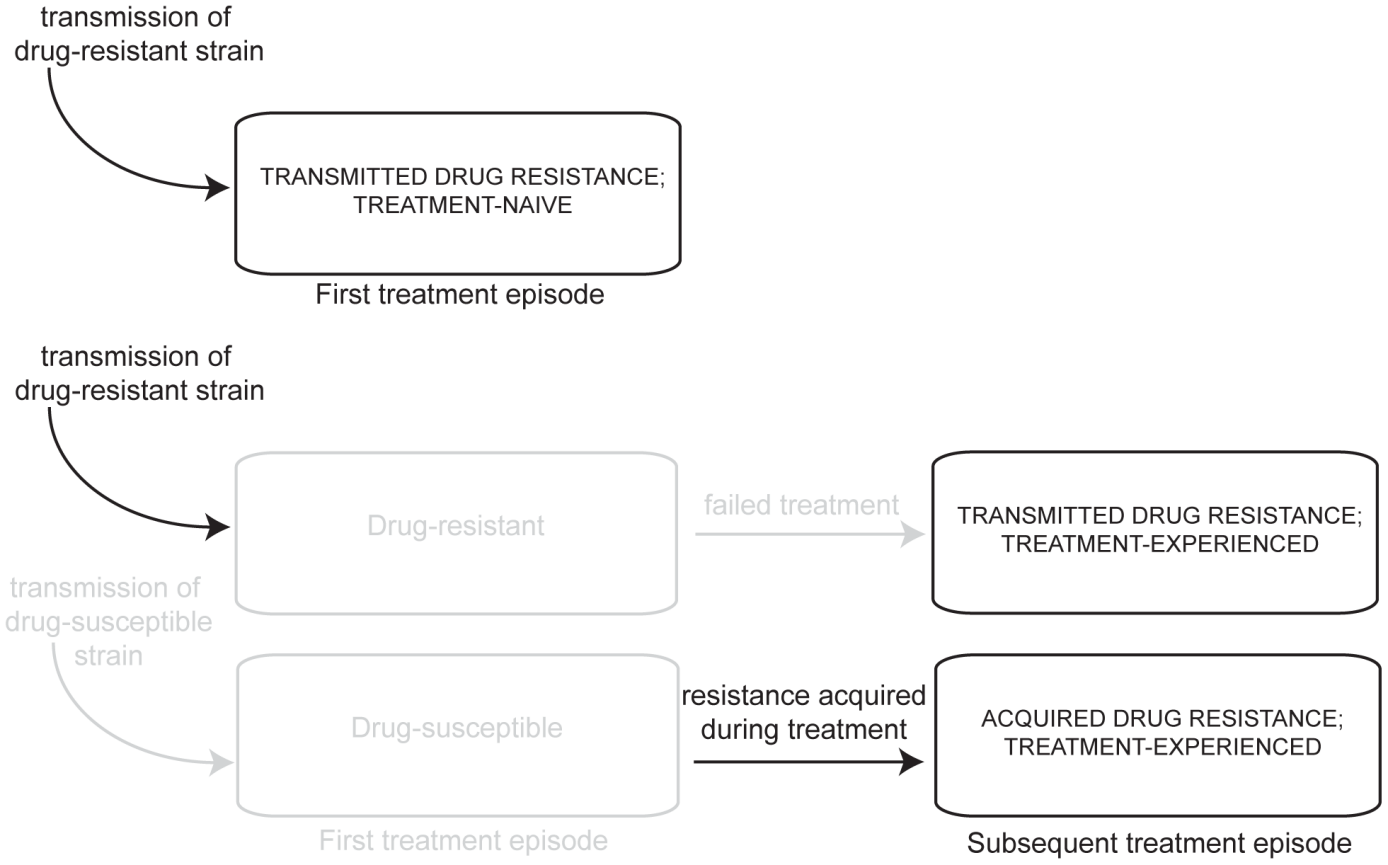
Mechanisms of TB drug resistance in treatment-naïve and experienced patients. The first pathway describes patients who test positive for resistance to anti-TB drugs prior to their first treatment episode. These treatment- naïve patients were initially infected with a drug-resistant strain. The second pathway describes patients who were infected by a drug resistant strain and failed their first course of treatment. After their first course of treatment, they tested positive for resistance to anti-TB drugs. The final pathway describes patients who were infected by a drug susceptible strain and failed their first treatment episode because they acquired resistance via spontaneous mutation.

Figure 1 displays mechanisms leading to drug resistant TB infection in treatment-naïve and treatment-experienced patients. Drug-resistance in treatment-naïve TB patients reflects primary transmission of resistant strains; in contrast, drug-resistance in TB patients who have previously been treated with anti-TB antibiotics may reflect either transmitted resistance or resistance acquired during previous treatment. Resistant strains observed among treatment-naïve TB patients have demonstrated sufficiently high reproductive fitness to have been transmitted and caused disease. By contrast, resistant strains that are observed among treatment-experienced patients arise from either transmission from another host or from within-host selection of sporadically occurring mutants under drug pressure. Drug resistant strains arising as a result of this second mechanism may not be as easily transmitted to secondary hosts as drug strains that have already demonstrated their ability to infect and cause disease in secondary hosts. Determining which strains are sufficiently fit to be transmitted and cause disease can aid in developing effective strategies to combat the spread of resistance.

Probabilistic graphical models, e.g. branching tree mixture models, have been used to infer the sequence of several binary events that have occurred in an unknown order [1]–[3]. These models can potentially provide public health benefit as they only require cross-sectional data, often easily and abundantly available, and are applicable to any biological system that follows an ascending Markov process. Past use of these models include describing the order of acquiring copy number aberrations in renal cancer, modeling the development of HIV genetic mutations associated with antiretroviral resistance and characterizing the acquisition of anti-TB drug resistance from phenotypic TB data [1]–[3]. Knowledge regarding these longitudinal processes may be useful in directing research for disease control.

Considerable work has been done in defining and fitting branching tree models. The single mutagenetic tree introduced by Desper et al. [1], describes the progression of a set of events, or pathway, for a population. The model assumes that there are no reversions following an event and that for each event, there is a unique pathway leading to it. To broaden this class of models for settings where the latter assumption does not hold, Beerenwinkel et al. [2] introduced mixture models that allow for the existence of multiple evolutionary pathways leading to the same event. Izu et al. [3] developed a Bayesian approach to identifying a mixture model and estimating the associated parameters.

Branching trees are useful in the context of TB because the probability of reverse mutations is very small (validating the ascending markov assumption), and global cross-sectional phenotypic drug resistance data are publicly available [4]. In analyses of genetic data, an event is a specific mutation; whereas in analyses of phenotypic data, sets of genetic mutations are grouped into single events. For example, the event “resistance to isoniazid” would comprise all patterns of genetic mutations which confer isoniazid resistance. Although phenotypic data does not allow examination of the ordering in which such mutations emerge, such data are more readily available and can provide a basis for generating hypotheses that can subsequently be tested with genetic data.

Below, we expand the use of these models beyond their previous application for describing the progression of events in a single population. This paper develops a Bayesian approach to compare pathways in two different populations using branching trees in which some tree parameters are prespecified. We apply these methods to investigate the relationship between drug resistance in treatment-naïve and in treatment-experienced patients. By comparing branching trees from these two groups of patients, we gain insight into the evolution of highly drug-resistant strains that remain capable of being transmitted and causing secondary disease.

## Methods

### Branching trees

**Figure 2:**
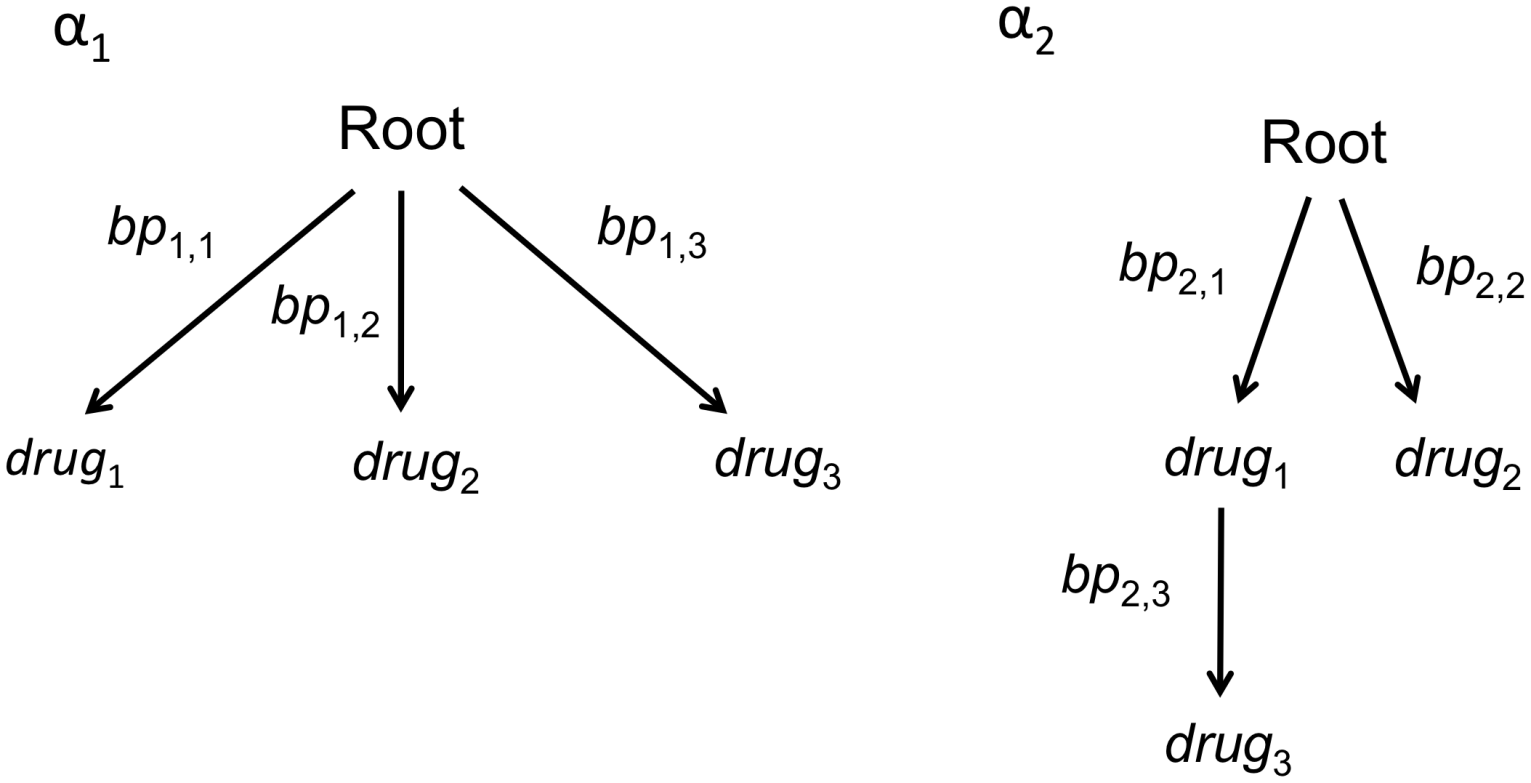
An example of the graphical display of a 2-tree mixture model with three nodes. 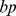
 is the set of edge weights defined as the conditional probability of the child node given the prior occurrence of the parent event. 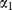 and 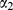 are the probability that an individual follows a pathway represented by the first and second tree, respectively.

We follow Desper et al. in our notation for branching tree models. A branching tree, denoted by 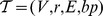, is a special Bayesian network that consists of a set of nodes or vertices, a root, a set of edges connecting the vertices, and edge weights. Vertices represent the event of a binary random variable and the root represents the binary random variable indicating whether none or at least one of the events characterized by the vertices have occurred. The edges connecting the nodes have weights equal to the conditional probability of the child event given the prior occurrence of the parent event. As the branching trees described here do not take time into account, the edge weights are not informative about the times to occurrences of events. An example is provided by the two trees in Figure 2 with edge weights 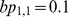 and 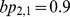. From these, we infer that prevalence of 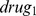 is higher but not that resistance to it occurs faster in the latter compared with the former tree. For more details on timed branching tree used in oncogenesis see Desper et al.

Branching trees model the joint distributions of events and impose constraints on the dependencies among events and on the order in which they can occur. Let 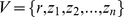 be the set of nodes for which 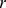 is the root; 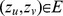 denote the edge directed from node 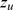 pointing towards node 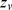 ;and 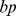 be the probability mapping such that 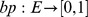. A path from 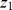 to 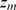 is a sequence of edges 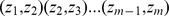 and 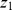 is an ancestor of 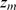. The path is a cycle if 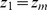. A branching tree imposes the restriction that there be no cycles and that each edge must be directed toward a different node. A node with no offspring is called a leaf. One particular branching tree to define is a star tree.

In this paper, the nodes represent the acquisition of drug resistance to one or more drugs and the root represents a wild type state (i.e. full sensitivity to all anti-TB drugs). The edges connecting the nodes signify that the event represented by the offspring (child) node can only occur given the prior occurrence of the event represented by the parent node. The edge weights are the conditional probabilities of these events.

### Mixture models

Because a branching tree requires that each edge be directed toward a different node, single branching trees may not be sufficient to describe the underlying processes of interest. Beerenwinkel et al. [2] introduced mixture models in order to accommodate the existence of multiple evolutionary pathways leading to the same node. A 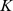-tree mixture model is comprised of 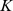 branching trees, 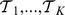, and their respective tree weights, 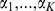, where 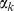 is the marginal probability that a random individual follows a pathway represented by the 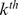 tree. Let 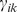 denote the probability that the 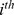 individual follows a pathway represented by 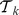 (Beerenwinkel et al. [2] referred to this probability as the *responsibility* of 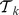). We refer to a tree structure as the graph of the mixture model without the edge weights, i.e. the collection of trees, 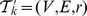.

Mixture models often contain a special noise component or star tree, in which all nodes originate in the root. Figure 2 provides an example of a mixture model, in which the first tree is the star tree. Mixture models which include a star tree ensure that every possible multinomial state has probability greater than zero.

### Estimating mixture models that are partially specified

We adapt the two-step process introduced in Izu et al. to estimate mixture models, in which aspects are prespecified
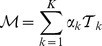
where 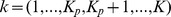 and 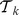 is treated as known for 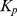 of the K trees. The first step estimates the structures of the remaining trees. To accomplish this, we adjust the EM-like algorithm in Beerenwinkel et al. [2] to account for the prespecified portion of the model. This involves iterating between estimating the 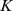 responsibilities for each individual and reconstructing the remaining 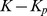 trees using the data weighted by the responsibilities. Given an estimate of 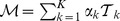 the responsibility of the 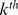 tree for the 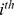 sample is estimated (E step) by
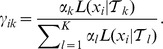
Following this step, 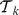 for 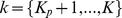 is reconstructed by using the maximum branching algorithm (M step) found in Desper et al. with the following adjusted joint and marginal probabilities
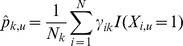


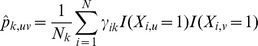


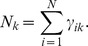
As discussed in Izu et al. we can also compose a set of candidate models that include similar, but different, structures for the unspecified trees and then use a given criteria to choose the best model. In certain settings, it may be reasonable to assume the structure of all trees in the model thereby avoiding the need for the first step.

Given the structure of the 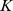 trees, the second step uses Bayesian methods to estimate the parameters associated with the partially-known mixture model. Let 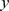 represent a multinomial random variable whose outcomes are determined by the pattern of events for the set of binary random variables or vertices. There are 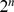 possible outcomes, where n is the number of vertices. Let 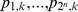 be the corresponding probabilities of each outcome associated with the 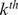 tree. For example, for the mixture model shown in Figure 2 there are 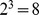 possible outcomes for the multinomial distribution. If 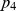 corresponds to the event resistance to 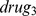 but not 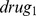 or 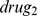, the probability of this outcome is
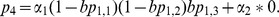
Let 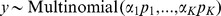. We place non-informative priors on the tree weights, 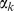, and the parameters associated with 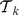. The posterior distribution of these parameters can be obtained from an MCMC implementation in WinBUGS.

### Measure of similarity

To use mixture models to compare two populations, A and B, we include trees derived from data on population A as prespecified elements in our mixture model for population B. Tree weights associated with these trees provide a measure of the similarity between the two populations, which we define below. The mixture model for population B is
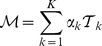
where 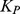 of the 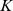 trees describe pathways that are also seen in population A, and the remaining trees describe pathways seen only in population B. We define the measure of similarity as 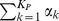. From our definition of 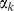 above, the measure of similarity is the probability that an individual from population B follows any of the pathways resulting from the model describing population A. Using the bayesian methods described above, we can obtain a posterior distribution for this quantity.

### Application to drug resistant TB in treatment-naïve and treatment-experienced patients

The data we analyze are obtained from Anti-Tuberculosis Drug Resistance in the World, Fourth Global Report [4]. These data arise from surveillance in countries where all notified culture-positive TB cases received drug susceptibility testing (DST) and from population-representative surveys in countries where not all TB cases routinely receive DST. Between 1994 and 2007, DST results were collected from patients from 138 settings in 114 countries and 2 Special Administrative Regions (SARs) of China. The anti-TB drugs reported include isoniazid (H), rifampin (R), ethambutol (E) and streptomycin (S). Twenty-nine settings were excluded because data were either only reported for treatment-naïve patients or combined for naïve and treatment-experienced patients, leaving a total of 85,672 samples from treatment-naïve patients and 18,619 samples from treatment-experienced patients. Seven different regions were considered (AFR = African region, AMR = region of the Americas, EMR = Eastern Mediterranean region, FSU = Former Soviet Union region, NFSU-EUR = Non-Former Soviet Union European region, SEAR = South-East Asian region, WPR = Western Pacific region) as shown in Table 1. Originally, all European countries were included in a single region. However, the prevalence of resistance to any anti-TB drug is significantly higher in countries of the former Soviet Union than in other European countries: 39% (95% CI: 38–40) and 8.2%(95% CI: 7.8, 8.5), respectively, among treatment-naïve cases and 71% (95% CI: 70–72) versus 20% (95% CI: 18, 22) among treatment experienced cases. Because of this large difference, we split the European region into two sub-regions.

**Table 1:** Breakdown of data by region.

	AFR	AMR	EMR	FSU	NFSU-EUR	SEAR	WPR
naïve	n = 13229	n = 12286	n = 2642	n = 7546	n = 21585	n = 4781	n = 23603
	0.11	0.15	0.15	0.39	0.08	0.15	0.18
	(0.11,0.12)	(0.14,0.15)	(0.14,0.16)	(0.38,0.40)	(0.08,0.09)	(0.14,0.16)	(0.17,0.18)
experienced	n = 2357	n = 2861	n = 511	n = 5335	n = 2461	n = 1553	n = 3541
	0.21	0.29	0.47	0.71	0.2	0.43	0.44
	(0.2,0.23)	(0.27,0.31)	(0.42,0.51)	(0.7,0.72)	(0.19,0.22)	(0.40,0.45)	(0.43,0.46)

The first row displays sample size, proportion of the population resistant to any drug and the corresponding confidence interval for treatment-naïve patients and the second row displays this information for patients with a previous treatment history. AFR = African region, AMR = region of the Americas, EMR = Easter Mediterranean region, FSU = Former Soviet Union region, NFSU-EUR = Non-Former Soviet Union European region, SEAR = South-East Asian region, WPR = Western Pacific region.

Resistance pathways may vary between regions, both because of geographic heterogeneity in strain lineage and because of differential selective pressure due to different historic usage of anti-tuberculosis drugs [5]. As a consequence, we analyze data from each region separately. Methods described in Izu et al. are used to analyze the data from the treatment-naïve patients. The resulting tree structures and their corresponding edge weights comprise the prespecified components in the mixture model fit to data from treatment-experienced patients.

## Results

### Results for treatment-naïve patients

**Figure 3:**
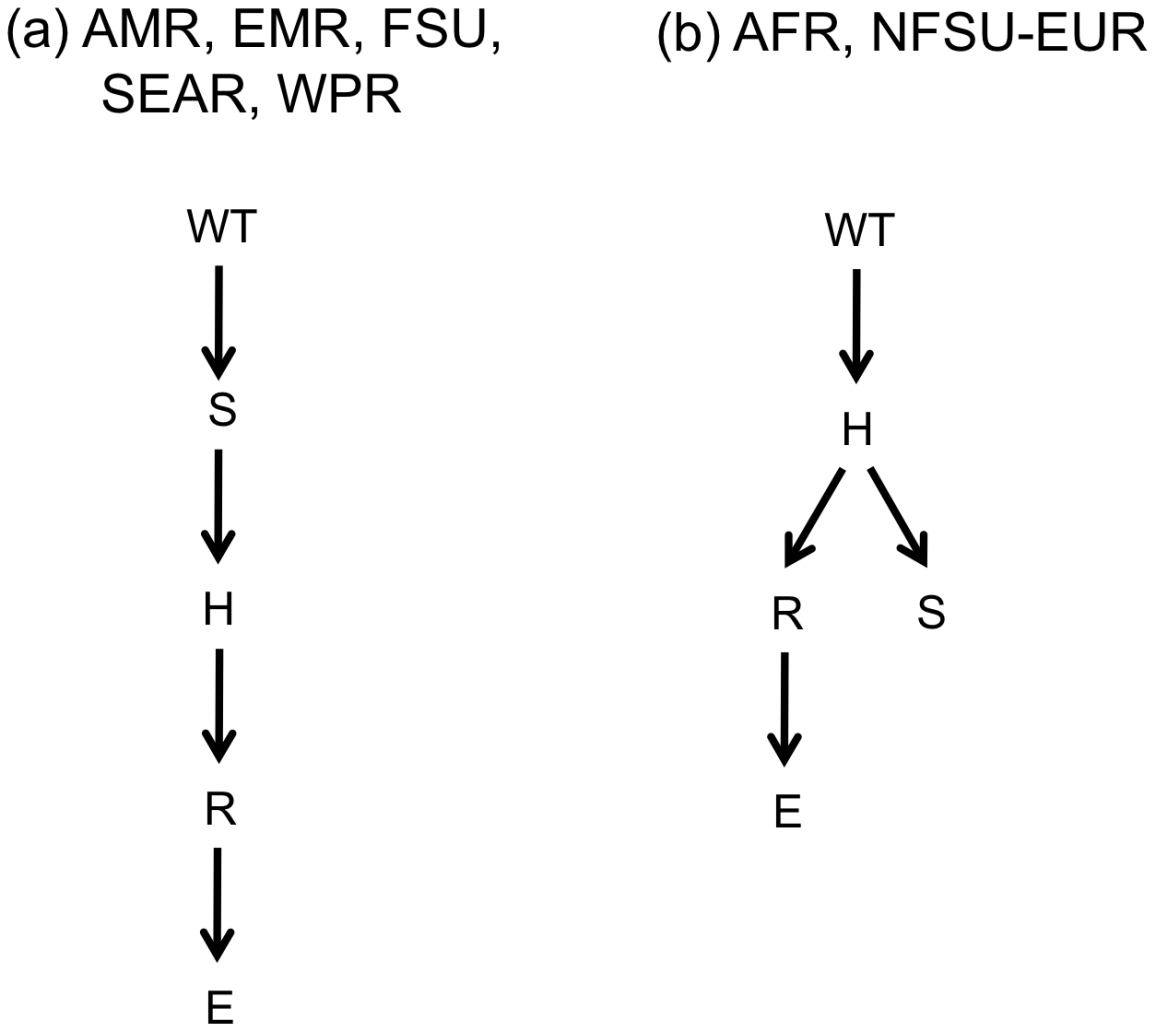
Non-star tree structures from mixture models for treatment-naïve patients. (a) non-star tree for AFR and NFSU-EUR. (b) non-star tree for AMR, EMR, FSU, SEAR and WPR. Nodes = {WT = wild type, H = isoniazid, R = rifampin, E = ethambutol and S = streptomycin }.

In the resulting mixture models for treatment-naïve patients, models from all seven regions contain two trees. The non-star tree for the models describing the AMR, EMR, FSU, SEAR and WPR is shown in Figure 3(a)-these are all trees with a single leaf. Izu et al. used a simulation study to show that these methods perform well when the underlying data generating tree structure has a single leaf. The non-star tree from the models describing AFR and NFSU-EUR is shown in Figure 3(b). For each region, estimates for the tree and edge weight parameters are shown in Table 2. 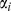 is an estimate of the proportion of the population following the 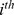 tree. The four columns following 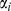 represent the edges and corresponding edge weights associated with tree 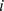. The edge weight is the conditional probability of resistance to the drug indicated by the child node given resistance to the drug indicated by the parent node. If the parent node is the root (WT), the edge weight is the marginal probability of resistance to the drug indicated by the child node. For example, in the AFR, 16% of all TB strains follow pathways described by the first tree which has the set of edges E = {WT
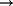
H, WT
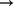
R, WT
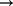
E, WT
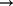
S}. 84% of TB strains follow pathways to resistance described by the second tree with the set of edges E = {WT
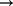
H, H
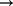
R, H
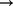
E, R
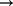
S}. In the latter, the conditional probability of resistance to rifampin given resistance to isoniazid is 0.86. The weights on the star tree found in the first column of Table 2, range from 0.09 (SEAR) to 0.18 (FSU) and all standard errors are less than 0.026. With the exception of the FSU, the probabilities associated with edges beginning at the root in the non-star tree are all less than 0.10 (s.e. 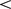
0.015), reflecting the relatively low prevalence of resistance observed among treatment-naïve patients. In contrast, for the FSU, the probability associated with the edge from the root is 0.27 (s.e. = 0.007).

**Table 2:** Posterior nodes and standard deviations of mixture model parameters describing resistance in treatment-naïve patients.

Region	Mixture Model Parameters									
AFR	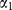	WT 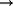 H	WT 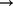 R	WT 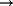 E	WT 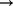 S	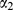	WT 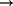 H	H 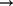 R	R 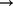 E	H 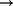 S
	0.16(0.007)	0.32(0.022)	0.047(0.006)	0.04(0.005)	0.35(0.020)	0.84(0.007)	0.02(0.003)	0.86(0.094)	0.51(0.043)	0.70(0.039)
AMR	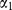	WT 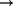 H	WT 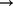 R	WT 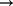 E	WT 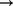 S	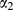	S 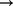 H	H 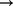 R	R 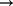 E	WT 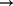 S
	0.11(0.006)	0.45(0.026)	0.19(0.015)	0.11(0.010)	0.20(0.025)	0.89(0.006)	0.32(0.021)	0.46(0.039)	0.46(0.049)	0.079(0.004)
EMR	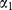	WT 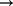 H	WT 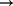 R	WT 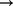 E	WT 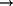 S	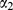	S 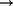 H	H 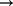 R	R 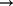 E	WT 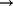 S
	0.13(0.026)	0.27(0.052)	0.17(0.038)	0.09(0.023)	0.32(0.057)	0.87(0.026)	0.42(0.079)	0.42(0.077)	0.78(0.091)	0.08(0.014)
FSU	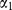	WT 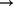 H	WT 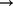 R	WT 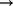 E	WT 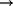 S	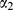	S 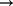 H	H 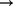 R	R 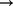 E	WT 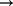 S
	0.18(0.007)	0.79(0.015)	0.29(0.016)	0.36(0.016)	0.38(0.022)	0.82(0.007)	0.64(0.013)	0.58(0.019)	0.62(0.023)	0.27(0.007)
NFSU-EUR	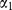	WT 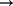 H	WT 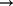 R	WT 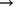 E	WT 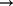 S	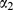	WT 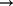 H	H 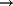 R	R 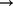 E	H 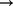 S
	0.13(0.005)	0.35(0.018)	0.047(0.006)	0.03(0.004)	0.26(0.014)	0.87(0.005)	0.01(0.001)	0.92(0.065)	0.58(0.048)	0.69(0.044)
SEAR	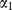	WT 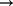 H	WT 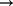 R	WT 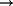 E	WT 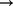 S	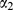	S 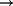 H	H 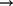 R	R 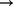 E	WT 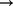 S
	0.09(0.008)	0.71(0.044)	0.17(0.023)	0.12(0.018)	0.28(0.046)	0.91(0.008)	0.25(0.048)	0.69(0.130)	0.68(0.090)	0.08(0.007)
WPR	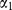	WT 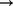 H	WT 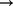 R	WT 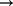 E	WT 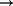 S	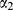	S 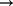 H	H 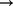 R	R 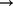 E	WT 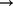 S
	0.11(0.004)	0.67(0.016)	0.27(0.011)	0.16(0.009)	0.28(0.019)	0.89(0.004)	0.34(0.017)	0.38(0.025)	0.65(0.048)	0.10(0.003)

The four columns following 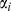 represent the edges and corresponding edge weights associated with tree i. The edge weight is the conditional probability of being resistant to the child node given resistance to the parent node has occurred. If the parent node is the root (WT), the edge weight is the marginal probability of becoming resistant to the child node. Nodes = {WT = wild type, H = isoniazid, R = rifampin, E = ethambutol and S = streptomycin }.

### Results for treatment-experienced patients

**Figure 4:**
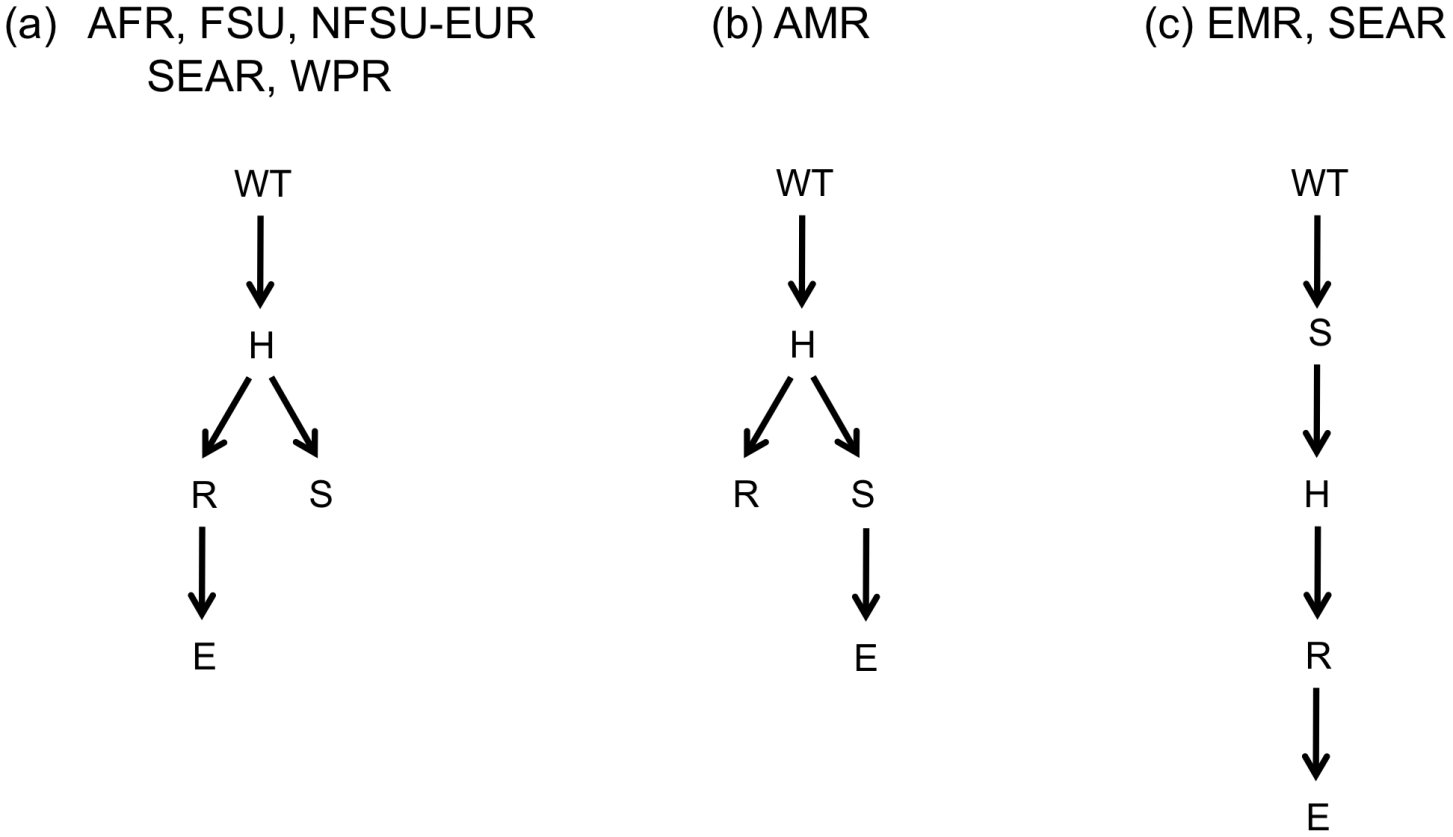
Non-star tree structures from mixture models for treatment-experienced patients. (a) non-star tree for AFR, FSU, NFSU-EUR, SEAR and WPR. (b) non-star tree for AMR (c) non-star tree for EMR and SEAR.

A prespecified mixture model was fit to the data on treatment-experienced patients with the non-star trees from the fit to data on naïve patients as specified components (Figure 3). The number of unspecified trees was obtained from fitting a fully specified mixture model to the data from treatment-experienced patients. The trees represented exclusively in the model for treatment-experienced patients describe pathways for resistance that are unique to this population (i.e. not observed among the treatment-naïve). Models for each region, with the exception of SEAR, contain two unspecified trees, one of which is the star tree, and the other of which is shown in Figure 4. The model describing the SEAR contains three unspecified trees: the star tree, and the trees shown in Figure 4(a) and 4(c). Each of the three different non-star tree structures, contain the edge H
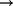
R. The non-star tree for the EMR and SEAR, is the only structure in which streptomycin, not isoniazid, is the child node of the root. The analysis of resistance patterns from treatment-naïve and experienced patients produces identical tree structures for the AFR, EMR, NFSU-EUR and SEAR.

The results of analyses are shown in Table 3. Because there is only one prespecified tree, the measures of similarity is the weight for the unspecified tree shown in the first column of Table 3. In our application, the measure of similarity is the probability that a treatment-experienced patient follows a pathway identical to that seen in treatment-naïve patients. It ranges from 0.29 to 0.71 and all standard errors are less than 0.18. The breakdown for each region is as follows: 0.52 (AFR, s.e. = 0.18), 0.71 (AMR, s.e. = 0.03), 0.36 (EMR, s.e. = 0.12), 0.33 (FSU, s.e. = 0.03), 0.48 (NFSU-EUR, s.e. = 0.18), 0.29 (SEAR, s.e. = 0.12), and 0.51 (WPR, s.e. = 0.06).

**Table 3:** Posterior nodes and standard deviations of mixture model parameters describing resistance in treatment-experienced patients.

Region	Tree weights comprising measure of similarity	Mixture model parameters from trees unique to treatment-experienced patients									
AFR	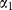	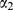	WT 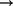 H	WT 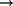 R	WT 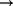 E	WT 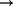 S	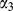	WT 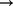 H	H 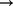 R	R 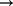 E	H 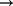 S
	0.52(0.18)	0.20(0.015)	0.47(0.053)	0.13(0.029)	0.062(0.012)	0.35(0.036)	0.27(0.18)	0.34(0.240)	0.84(0.097)	0.57(0.079)	0.68(0.065)
AMR	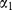	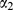	WT 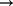 H	WT 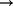 R	WT 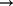 E	WT 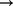 S	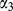	WT 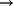 H	H 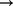 R	S 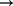 E	H 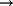 S
	0.71(0.032)	0.20(0.011)	0.62(0.032)	0.47(0.031)	0.11(0.014)	0.21(0.029)	0.088(0.030)	0.69(0.190)	0.94(0.033)	0.59(0.058)	0.95(0.045)
EMR	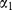	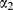	WT 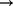 H	WT 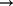 R	WT 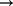 E	WT 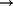 S	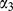	S 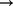 H	H 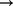 R	R 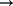 E	WT 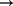 S
	0.36(0.120)	0.24(0.028)	0.58(0.069)	0.58(0.069)	0.22(0.045)	0.55(0.066)	0.40(0.120)	0.87(0.055)	0.95(0.034)	0.78(0.073)	0.58(0.160)
FSU	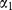	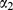	WT 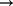 H	WT 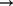 R	WT 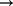 E	WT 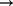 S	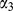	WT 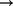 H	H 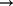 R	R 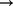 E	H 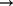 S
	0.33(0.034)	0.24(0.014)	0.67(0.028)	0.31(0.031)	0.25(0.020)	0.57(0.020)	0.43(0.032)	0.93(0.048)	0.97(0.018)	0.74(0.015)	0.71(0.011)
NFSU-EUR	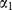	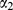	WT 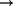 H	WT 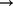 R	WT 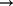 E	WT 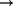 S	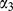	WT 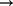 H	H 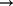 R	R 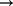 E	H 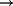 S
	0.48(0.180)	0.18(0.010)	0.55(0.039)	0.19(0.030)	0.10(0.016)	0.34(0.031)	0.34(0.180)	0.25(0.190)	0.97(0.030)	0.64(0.055)	0.85(0.045)
SEAR	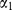	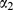	WT 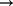 H	WT 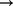 R	WT 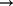 E	WT 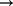 S	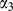	S 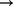 H	H 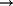 R	R 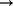 E	WT 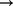 S
	0.29(0.12)	0.22(0.033)	0.92(0.027)	0.38(0.091)	0.11(0.045)	0.37(0.057)	0.25(0.100)	0.50(0.200)	0.80(0.200)	0.76(0.220)	0.50(0.210)
		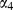	WT 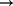 H	H 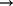 R	R 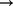 E	H 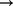 S					
		0.24(0.099)	0.40(0.210)	0.66(0.260)	0.74(0.180)	0.47(0.270)					
WPR	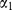	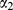	WT 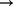 H	WT 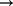 R	WT 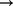 E	WT 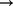 S	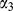	WT 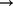 H	H 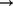 R	R 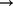 E	H 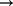 S
	0.51(0.056)	0.30(0.016)	0.67(0.029)	0.46(0.033)	0.12(0.014)	0.41(0.020)	0.19(0.053)	0.68(0.160)	0.93(0.049)	0.89(0.069)	0.60(0.028)

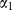
 corresponds to the tree weights of the prespecified trees. Nodes = {WT = wild type, H = isoniazid, R = rifampin, E = ethambutol and S = streptomycin }.

As shown in Izu et al., bootstrap methods provide information regarding the stability of these tree structures. For each region, a set of candidate tree structures are obtained for naïve and treatment-experienced patient from fitting 30 bootstrap samples. The program Mtreemix [6] was used to fit Beerenwinkel's mixture model to data from naïve patients and an adaption to the Mtreemix program was used to fit our prespecified mixture model to data from treatment-experienced patients. All candidate sets contain fewer than four structures with the exception of the NFSU-EUR and SEAR for treatment-experienced patients (five and eight structures, respectively). Results of Izu et al. imply that estimates from models where more structures occur in the set of candidate trees are less stable. Results provided in Table 3 show that the standard deviations of the posterior distribution for the branching tree parameters in these regions are relatively high.

### Simulation study

In analyses described above, we prespecified a single tree in our mixture model. This section presents the results of simulations to gauge the accuracy of our methods. Data are simulated from the seven resulting mixture models from the treatment-experienced data. In each of the models, labeled simulations 1–7, one tree structure and its edge weights are prespecified and treated as known. We estimate the structure and corresponding edge weights for the remaining unspecified portion of the model as well as all tree weights.

Table 4 shows how often the correct tree structure is chosen. The agreement between these results and those from the bootstrap analyses (Table 5) are generally high, with some notable exceptions. The results from AFR, EMR, and WPR appear to be stable in both analyses and the results for SEAR are particularly unstable in both. In the AMR, FSU and NFSU-EUR, the results from the simulation samples differ from the results from the bootstrap samples. The NFSU-EUR shows the largest difference. The correct tree structure is chosen in 84% of the simulations, but in only 3% of the bootstrap samples. The tree structure chosen in 83% of the bootstrap samples is similar to the correct tree except for the non-star tree in the unspecified portion of the model. The set of edges for the non-star tree is: E = {WT
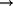
H, H
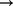
R, H
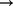
E, H
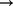
S}. We compared the distribution of the bootstrap samples that resulted in this alternative tree and the simulation samples resulting in the correct tree. Eight of the sixteen multinomial parameters show different distributions in the bootstrap compared to the simulation samples. We believe that these differences constitute the main driver of this discrepancy. Such differences could make it difficult for the data to distinguish between closely related trees (e.g. those that differ by a single edge) that explain the data equally well.

**Table 4:** The percentage of simulations in which the correct tree structure is chosen.

Simulation	Percentage
1 (AFR)	98.6
2 (AMR)	70.5
3 (EMR)	77.3
4 (FSU)	100
5 (NFSU-EUR)	84.2
6 (SEAR)	39.8
7 (WPR)	100

The region simulated is shown in parenthesis.

**Table 5:** Number of different structures that arose from 30 bootstrap samples fit to naïve and treatment-experienced patients in each region.

	AFR	AMR	EMR	FSU	NFSU-EUR	SEAR	WPR
naïve	4 (53)	1(100)	2(77)	2(07)	2(57)	1(100)	1 (100)
experienced	3(87)	4(40)	3 (37)	1(100)	5(2)	8(20)	1(100)

The number inside the parenthesis is the percentage of structures which were the same as that of the original sample.

The results from fitting the models are shown in Table 6, which provides the coverage for each parameter estimated in the seven models. Coverage is defined as the percentage of time the 95% credible intervals contain the true parameter, given the simulation resulted in the correct tree structure. Of all seven simulations, all parameter estimates have coverage higher than 90%. Our simulations show that when the tree structure is correct, the mixture model parameters are well estimated.

**Table 6:** Coverage for seven different simulations.

Region	Tree weights comprising measure of similarity	Mixture model parameters from trees unique to treatment-experienced patients									
1 (AFR)	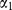	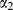	R 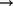 1	R 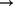 2	R 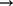 3	R 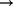 4	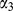	R 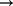 1	1 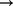 2	2 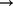 3	1 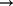 4
	100	97	97.2	95.2	95.2	97.2	100	100	97.4	95.8	94.3
2 (AMR)	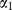	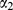	R 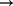 1	R 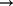 2	R 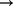 3	R 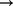 4	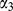	R 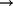 1	1 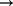 2	4 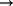 3	1 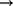 4
	92.5	95.5	92.8	93.2	95.3	92.3	91.2	95.6	95.6	96	95.9
3 (EMR)	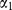	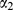	R 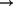 1	R 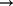 2	R 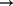 3	R 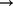 4	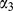	4 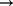 1	1 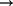 2	2 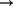 3	R 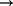 4
	100	97.8	96.8	95.1	95.3	97.4	100	97.2	97.7	96.1	100
4 (FSU)	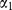	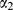	R 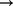 1	R 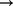 2	R 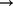 3	R 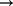 4	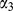	R 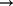 1	1 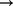 2	2 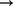 3	1 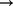 4
	99.4	98.3	95.4	96.6	97.1	96.9	98.7	99.4	96.8	94.6	95.4
5 (NFSU-EUR)	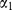	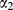	R 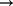 1	R 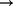 2	R 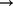 3	R 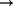 4	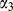	R 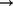 1	1 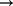 2	2 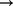 3	1 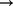 4
	100	95.6	95.6	94.5	92.8	92.5	100	100	95.4	95.5	93.9
6 (SEAR)	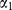		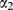	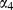	R 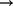 1	R 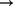 1	R 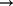 2	1 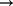 2	R 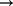 3	2 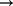 3	R 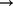 4
	100	98	95.5	99.5	96.7	97.5	100	100	99.7	99.5	99.7
	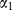		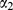	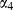	R 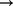 1	R 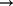 1	R 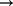 2	1 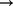 2	R 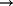 3	2 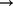 3	R 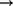 4
		100	100	99.7	100	99.5					
7 (WPR)	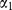	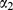	R 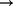 1	R 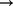 2	R 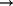 3	R 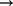 4	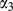	R 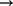 1	1 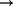 2	2 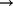 3	1 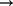 4
	96.8	96.9	94.5	96.5	96.3	95.9	94.4	98.4	97.5	96.1	93.8

Coverage is defined as the percentage of time the 95% credible intervals contain the true parameter, given the simulation resulted in the correct tree structure. R = root of the tree. Nodes = {1,2,3,4}. The region simulated is shown in parenthesis. The nodes {1,2,3,4} correspond to the drugs in the application: {H, R, E, S}. 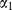 is the tree weight of the prespecified structures.

## Discussion

This paper describes methods to estimate partially prespecified mixture models which can be used to compare two populations. Our model is applied to investigate patterns of resistance amongst treatment naïve and experienced patients. Trees from treatment-naïve data (Figure 3) reflect pathways from strains which have demonstrated the ability to be transmitted and cause disease. Trees from treatment-experienced patients (Figure 4) describe pathways from a combination of transmitable and reproducible strains and those which may have suffered some cost in terms of their ability to transmit. There are different explanations for the patterns we observe in the two populations and these methods cannot definitively differentiate among them. Below, we review our results and use them to generate hypotheses about underlying mechanisms of TB resistance which may be worthy of further testing.

In the AFR, EMR, NFSU-EUR and SEAR the same tree structure arises from both treatment-naïve and experienced patients, implying the pathways to multi-drug resistance are similar in both populations. One possible explanation is that in these regions, all pathways result in transmissible resistant TB strains. Factors that are region specific provide other possible explanations. For example, there is a high prevalence of HIV in the AFR. Patients with suppressed immune systems may be more susceptible to strains that have lower overall reproductive fitness, thereby permitting all pathways observed among re-treatment cases to be also seen in naïve cases [7]. The NFSU-EUR has the lowest prevalence of drug resistance among all regions (Table 1). For both naïve and experienced patients in this region, much highly resistant disease is observed among immigrants from areas where the prevalence of drug resistance is high [8]. One potential explanation is that the majority of highly resistant disease in this region results from transmission with only minimal contribution of acquired resistance.

In contrast, analysis of AMR, FSU, SEAR and WPR resulted in branching trees which differ among treatment-naïve and experienced patients. This tends to imply that some pathways to resistance produce strains that are relatively less transmissible and cause disease in secondary hosts. Alternatively, it may be that new resistance pathways appearing first among re-treatment cases through acquisition may not have had enough time to be observed among new cases.

Among treatment-naïve patients, the pathway of the most common tree begins with streptomycin; however, in treatment-experienced patients, the majority of the trees, it begins with isoniazid. This difference may reflect the history of TB treatment. Streptomycin was the first anti-TB drug in general use followed by isoniazid and then rifampin. It is also possible that in some settings (and with some resistance-conferring mutations), resistance to isoniazid is associated with a reproductive fitness cost that decreases the microbes transmissibility or ability to cause disease [9]–[11]. It is unlikely that this ordering of mutations reflects current sequencing of drug use since in most settings the vast majority of cases will be treated simultaneously with four drugs (rifampin, isoniazid, ethambutol and pyrazinamide) [12]. Only in rare settings is streptomycin (the only antibiotic of the four reported here that requires injection) used in first-line regimens for treating tuberculosis.

Each non-star tree describing both treatment-naïve and experienced patients contains the edge H
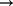
R. This important edge defines the development of multidrug resistant TB (MDR-TB). Given that a strain follows a pathway associated with the tree under study, the weight corresponding to the edge H
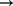
R is the conditional probability of the strain being MDR given that it is resistant to isoniazid (INH). This edge weight in the trees for naïve patients provides insight into the probability of MDR-TB given INH resistance in strains that are being transmitted. Except for the AFR, the H
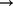
R edge weight is lower in trees associated with treatment-naïve patients, suggesting in these regions, the conditional probability of MDR-TB given INH resistance may be lower among transmitted strains.

The measure of similarity provides a quantitative measure of the degree of similarity of two populations. We note that it does not directly provide information regarding the process of acquiring resistance in the two populations. Resistance pathways seen in the sample of treatment- naïve patients may not actually represent every possible pathway associated with this population. In addition, patients presenting for re-treatment who were originally infected with resistant strains may also have acquired additional resistance [13]. Therefore, comparison of tree structures from treatment-naïve and treatment-experienced patients cannot serve as a basis for estimating the proportion of the latter who were originally infected with resistance strains. Nonetheless, the proportion of drug-resistant and MDR TB attributable to transmission found in several molecular epidemiologic studies, 38% to 53%, and 64% respectively are similar to the weights associated with trees observed in treatment-naïve patients [14]–[17].

The large amount of data from treatment-naïve patients allows us to estimate reliably the prespecified portion of the model. In some settings, it may not be appropriate to assume that branching trees are known for a portion of the model. The Bayesian approach permits incorporation of uncertainty by placing a prior distribution on the parameters of the prespecified trees; the methods of Szabo and Boucher [18] that permit incorporation of measurement error into the mixture model can also be used. We would have included this approach in our analysis had such measures been available in the settings where the data were collected. In other settings, it may be preferable to avoid prespecification of model components and estimate all model parameters completely from available data. To aid in such endeavors, our model could be naturally extended to include other covariates, such as indicator variables for different populations.

Izu et al. discuss the possibility that multiple structures may describe data equally well as was possibly the case in the NFSU-EUR. The authors recommend using bootstrap methods and simulation to assess reliability of results. In such situations, examining the similarities among the different plausible tree structures provides insight regarding resistance pathways. In the results described above, all of the trees resulting from the bootstrap samples shared many of the same properties. The most notable similarity was the role of E as the child node to R in 96.5% and 72.9% of the resulting structures from the bootstrap samples across all regions for naïve and experienced patients, respectively. 92.7% of the bootstrap samples across all regions for both groups of patients resulted in a structure with H as an ancestor to R, implying resistance to isoniazid precedes resistance to rifampin–a finding that has also been previously described.

In summary, the proposed methods permit investigation of pathways to resistance in treatment-naïve and treatment-experienced patients, subject to limitations describe above. These results are useful for formulating questions regarding the biology and epidemiology of drug resistant tuberculosis and can help generate testable hypotheses about which pathways to multiple drug resistance may be most likely to generate fit strains capable of being successfully transmitted. The analyses presented here are limited by the fact that only phenotypic resistance data were available. As discussed in Izu et al., genotypic data that permit inference regarding the pathways by which specific drug-resistance conferring mutations accumulate would allow for refinement of hypotheses that can be tested. Although the focus of this paper is on tuberculosis, our methods can be generalized to any biological process for which the assumption of an ascending markov process applies.
